# Supplementary material for: Admixture has obscured signals of historical hard sweeps in humans
Source: Nat Ecol Evol. 2022 Oct 31;6(12):2003–15. doi: 10.1038/s41559-022-01914-9 (PMC9715430; doi:10.1038/s41559-022-01914-9)
Supplement: Supplementary file 2 — Reporting Summary [file 41559_2022_1914_MOESM2_ESM.pdf]

nature portfolio

Corresponding author(s): YS, RT, AJ, AC, CDH

Last updated by author(s): 2022-08-26

Reporting Summary

Nature Portfolio wishes to improve the reproducibility of the work that we publish. This form provides structure for consistency and transparency in reporting. For further information on Nature Portfolio policies, see our Editorial Policies and the Editorial Policy Checklist.

Statistics

For all statistical analyses, confirm that the following items are present in the figure legend, table legend, main text, or Methods section.

n/a	Confirmed	
☐	☒	The exact sample size (*n*) for each experimental group/condition, given as a discrete number and unit of measurement
☐	☒	A statement on whether measurements were taken from distinct samples or whether the same sample was measured repeatedly
☐	☒	The statistical test(s) used AND whether they are one- or two-sided<br>*Only common tests should be described solely by name; describe more complex techniques in the Methods section.*
☐	☒	A description of all covariates tested
☐	☒	A description of any assumptions or corrections, such as tests of normality and adjustment for multiple comparisons
☐	☒	A full description of the statistical parameters including central tendency (e.g. means) or other basic estimates (e.g. regression coefficient) AND variation (e.g. standard deviation) or associated estimates of uncertainty (e.g. confidence intervals)
☐	☒	For null hypothesis testing, the test statistic (e.g. *F*, *t*, *r*) with confidence intervals, effect sizes, degrees of freedom and *P* value noted<br>*Give P values as exact values whenever suitable.*
☒	☐	For Bayesian analysis, information on the choice of priors and Markov chain Monte Carlo settings
☒	☐	For hierarchical and complex designs, identification of the appropriate level for tests and full reporting of outcomes
☐	☒	Estimates of effect sizes (e.g. Cohen's *d*, Pearson's *r*), indicating how they were calculated

Our web collection on statistics for biologists contains articles on many of the points above.

Software and code

Policy information about availability of computer code

Data collection	Data were manually curated from publicly available datasets.
Data analysis	Custom R and SLiM scripts used to perform analyses in this study are available from corresponding authors upon request.

For manuscripts utilizing custom algorithms or software that are central to the research but not yet described in published literature, software must be made available to editors and reviewers. We strongly encourage code deposition in a community repository (e.g. GitHub). See the Nature Portfolio guidelines for submitting code & software for further information.

Data

Policy information about availability of data

All manuscripts must include a data availability statement. This statement should provide the following information, where applicable:

- Accession codes, unique identifiers, or web links for publicly available datasets
- A description of any restrictions on data availability
- For clinical datasets or third party data, please ensure that the statement adheres to our policy

Access codes for all data used in the project are provided in the Data Availability statement. A complete description of how the raw sequence data were processed and prepared for analysis is provided in the Methods.

Field-specific reporting

Please select the one below that is the best fit for your research. If you are not sure, read the appropriate sections before making your selection.

- [ ] Life sciences
- [ ] Behavioural & social sciences
- [x] Ecological, evolutionary & environmental sciences

For a reference copy of the document with all sections, see nature.com/documents/nr-reporting-summary-flat.pdf

Ecological, evolutionary & environmental sciences study design

All studies must disclose on these points even when the disclosure is negative.

Study description	Population genomic analyses of hard sweep signals in ancient and modern human genomes with a focus on historical Eurasian populations. Putative hard sweeps were inferred in each tested population using SweepFinder2 method, with 57 candidate sweeps being determined in total. Additional analyses explore the evidence for hard sweep signal loss following known Holocene admixture events.
Research sample	Samples were chosen from all available Eurasian aDNA samples available at the commencement of the study and placed into 'populations' based on archaeological and population genetic criteria. The full rationale for the population groupings is described in Methods and SI.
Sampling strategy	Rationale for population groupings is described in Methods and SI.
Data collection	NA: study uses previously published data.
Timing and spatial scale	Rationale for population groupings is described in Methods and SI.
Data exclusions	Populations with small effective sample sizes were excluded from some analyses. The rationale is explained in the Methods and SI.
Reproducibility	All sequence processing and bioinformatic and statistical methods used in the study are reported in the Methods and SI.
Randomization	Rationale for population groupings is described in Methods and SI.
Blinding	NA: study uses previously published data.

Did the study involve field work? [ ] Yes [x] No

Reporting for specific materials, systems and methods

We require information from authors about some types of materials, experimental systems and methods used in many studies. Here, indicate whether each material, system or method listed is relevant to your study. If you are not sure if a list item applies to your research, read the appropriate section before selecting a response.

Materials & experimental systems

n/a	Involved in the study	
x		Antibodies
x		Eukaryotic cell lines
	x	Palaeontology and archaeology
x		Animals and other organisms
x		Human research participants
x		Clinical data
x		Dual use research of concern

Methods

n/a	Involved in the study	
x		ChIP-seq
x		Flow cytometry
x		MRI-based neuroimaging

Palaeontology and Archaeology

Specimen provenance	All data used in this study are sourced from publicly available datasets.
Specimen deposition	NA
Dating methods	Sample dates were obtained from published materials.

- [ ] Tick this box to confirm that the raw and calibrated dates are available in the paper or in Supplementary Information.

Ethics oversight	NA

Note that full information on the approval of the study protocol must also be provided in the manuscript.
